# Immunogenicity of botulinum toxins

**DOI:** 10.1007/s00702-012-0893-9

**Published:** 2012-09-25

**Authors:** Markus Naumann, Lee Ming Boo, Alan H. Ackerman, Conor J. Gallagher

**Affiliations:** 1 Medical Affairs, Allergan, Inc., 2525 Dupont Drive, Irvine, CA 92612 USA; 2 Department of Neurology, Klinikum Augsburg, Augsburg, Germany

**Keywords:** Botulinum neurotoxins, Neutralizing antibodies, Cervical dystonia, Torticollis, Blepharospasm, Hyperhidrosis

## Abstract

Botulinum neurotoxins are formulated biologic pharmaceuticals used therapeutically to treat a wide variety of chronic conditions, with varying governmental approvals by country. Some of these disorders include cervical dystonia, post-stroke spasticity, blepharospasm, migraine, and hyperhidrosis. Botulinum neurotoxins also have varying governmental approvals for cosmetic applications. As botulinum neurotoxin therapy is often continued over many years, some patients may develop detectable antibodies that may or may not affect their biological activity. Although botulinum neurotoxins are considered “lower risk” biologics since antibodies that may develop are not likely to cross react with endogenous proteins, it is possible that patients may lose their therapeutic response. Various factors impact the immunogenicity of botulinum neurotoxins, including product-related factors such as the manufacturing process, the antigenic protein load, and the presence of accessory proteins, as well as treatment-related factors such as the overall toxin dose, booster injections, and prior vaccination or exposure. Detection of antibodies by laboratory tests does not necessarily predict the clinical success or failure of treatment. Overall, botulinum neurotoxin type A products exhibit low clinically detectable levels of antibodies when compared with other approved biologic products. This review provides an overview of all current botulinum neurotoxin products available commercially, with respect to the development of neutralizing antibodies and clinical response.

## Introduction

Botulinum neurotoxins have been shown to be effective in the treatment of a variety of chronic conditions. For example, in the United States, onabotulinumtoxinA is approved for the treatment of cervical dystonia (CD), post-stroke spasticity of the upper limb, blepharospasm, strabismus, hyperhidrosis, chronic migraine, glabellar lines, and neurogenic detrusor overactivity (BOTOX® [package insert] 2011). Because many of the indications for botulinum neurotoxins are chronic conditions requiring long-term therapy, repeat botulinum neurotoxin treatments are typically required over a prolonged period of time. This may lead to the development of antibodies to botulinum neurotoxins, which are detectable by various tests although the antibodies may or may not affect the biological activity of the toxin. This review will explore the composition and immunologic potential of botulinum neurotoxins, the different types of antibodies that can be generated in response to botulinum neurotoxins, the methods used to detect these antibodies, and the factors that affect antibody formation. The paper will then review the clinical antibody data available for each botulinum neurotoxin product.

## Composition of natural BoNTs and BoNT products

The bacteria *Clostridium botulinum*, *Clostridium butyricum*, and *Clostridium baratii* together produce the seven different serotypes of botulinum neurotoxins found in nature (types A–G) (Poulain et al. 2008). Botulinum neurotoxins are transcribed by the bacteria as protein complexes consisting of a core neurotoxin and a number of associated non-toxic accessory proteins (NAPs). The core botulinum neurotoxin (BoNT) is a 150-kDa protein that consists of a 100-kDa heavy chain and a 50-kDa light chain, which are linked by a disulfide bond (Kukreja and Singh 2007). The NAPs are comprised of hemagglutinin and non-toxin, non-hemagglutinin proteins (Inoue et al. 1996) and spontaneously associate with the core neurotoxin (Poulain et al. 2008) following their co-synthesis by the bacteria. They have been shown to help stabilize and protect the core neurotoxin from changes in temperature, low pH, and enzymatic degradation (Brandau et al. 2007; Gu et al. 2012; Kukreja and Singh 2007).

Only two of the serotypes of botulinum neurotoxins (A and B) are used to formulate commercially available biologic products for clinical use. The type A botulinum neurotoxin products are onabotulinumtoxinA (BOTOX®; Allergan, Inc., Irvine, CA, USA), abobotulinumtoxinA (Dysport™; Ipsen Biopharm Ltd., Wrexham, UK), and incobotulinumtoxinA (Xeomin®; Merz Pharmaceuticals, Frankfurt am Main, Germany), whereas the type B botulinum neurotoxin is rimabotulinumtoxinB (Myobloc®; Solstice Neurosciences, LLC, South San Francisco, CA, USA, a wholly owned subsidiary of US WorldMeds, LLC, Louisville, KY, USA). All commercially available botulinum neurotoxin products contain the core BoNT and excipients (e.g., albumin) and all botulinum neurotoxin products, except for incobotulinumtoxinA, include NAPs, which are removed during the manufacturing of incobotulinumtoxinA (FDA Approval Package for Xeomin® 2010). Although lacking in NAPs, incobotulinumtoxinA is stabilized by virtue of its excipient composition. AbobotulinumtoxinA, onabotulinumtoxinA, and rimabotulinumtoxinA contain different complements of NAPs and, therefore, have different molecular sizes and three-dimensional structures (Krebs and Lebeda 2008).

## Antibodies against botulinum neurotoxins

Because commercially available botulinum neurotoxin preparations contain non-human proteins (excluding the excipient albumin), they may act as antigens and elicit antibody formation when injected into a patient. Two distinct types of antibodies may form after exposure to botulinum neurotoxin products: neutralizing and non-neutralizing. Neutralizing antibodies have been reported to form primarily against the heavy chain of the core BoNT; however, neutralizing antibodies that bind to epitopes on all regions of the core BoNT have been observed (Dolimbek et al. 2007; Atassi et al. 2011). If present in sufficient titers, these antibodies can inhibit the biological activity of the toxin, possibly by blocking its interaction with its neuronal receptor (Dolimbek et al. 2007; Atassi et al. 2008). In contrast, non-neutralizing antibodies are produced either against the NAPs or bind to the core BoNT, but they do not affect the biologic activity of the toxin and are not expected to interfere with the clinical efficacy of the product (Göschel et al. 1997).

## Immunogenicity versus non-response

It is important to distinguish between immunogenicity and the clinical classifications of secondary non-response and primary non-response. As described above, immunogenicity refers to the ability of a protein product to elicit antibody formation. Secondary non-response describes the situation where a patient initially responds to therapy but then loses clinical responsiveness over time with repeated treatments. In contrast, primary non-response occurs when a patient fails to respond to the first and any subsequent administration of a therapy. The former may be due to the formation of neutralizing antibodies; however, the presence of such antibodies does not always predict treatment non-response, since at least some patients with neutralizing antibodies retain normal sensitivity to botulinum neurotoxins (Carruthers et al. 2004; Brin et al. 2008; Muller et al. 2009). Conversely, many patients deemed clinically non-responsive do not have detectable neutralizing antibodies (Hanna and Jankovic 1998; Lange et al. 2009). In some cases, this may be due to the sensitivity of the test used to measure antibodies. A large study of secondary non-responders to botulinum toxin products (onabotulinumtoxinA or abobotulinumtoxinA) showed that less than half (44.5 %; 224/503) of patients were positive for neutralizing antibodies using the mouse hemidiaphragm assay (MDA) (discussed later), which indicates that in clinical practice factors other than immunogenicity may contribute to treatment non-response (Lange et al. 2009). Lack of clinical benefit can be caused by technical issues such as inadequate dosing, failure to accurately identify and inject the selected muscles contributing to the clinical syndrome being treated, or difficulty targeting the intended muscle (Brin et al. 2004). Changes in disease state over time and unrealistic patient expectations may impact the perceived success of repeated treatments (Brin et al. 2004).

## Factors affecting the immunogenicity of botulinum neurotoxins products

Many factors can influence the immunogenicity of biological therapeutics such as botulinum neurotoxins. These can include factors related to the product itself as well as factors related to treatment.

### Product-related factors

#### Manufacturing processes

Even small changes in the manufacturing process can alter the three-dimensional structure of therapeutic proteins, which can change their clinical performance as well as their immunogenicity. For example, the method of isolation, the method of finishing in the drying process, the type and/or amount of excipients present, or inadvertent contact with unprotected surfaces can lead to variability in the composition and/or structure of the final product and can alter its immunogenicity (Gottlieb 2008).

#### Toxin source

The source of the toxin can cause variations in immunogenicity. For example, the BoNT/A lot initially used in the manufacture of the first commercially available botulinum neurotoxin product (onabotulinumtoxinA; originally known as Oculinum) contained 25 ng of neurotoxin protein per 100 U (Jankovic et al. 2003), and the immunogenicity rate with this product was reported to be as high as 15 % (Jankovic and Schwartz 1991). In 1997, an updated bulk toxin source became available and since that time manufactured lots of onabotulinumtoxinA contain approximately 5 ng of neurotoxin per 100 U (Jankovic et al. 2003); this has been associated with at least a six-fold decrease in the rate of reported immunogenicity (Jankovic et al. 2003; Naumann et al. 2010).

#### Inactive toxin

As mentioned earlier, the 150-kDa core BoNT of botulinum neurotoxin products, which is initially produced by the bacteria in an inactive form, can be immunogenic. Therefore, the amount of inactive toxin in botulinum neurotoxin products should be kept as low as possible to limit the overall amount of core BoNT protein to that which can produce a therapeutic effect and thus decrease their antigenic potential. For this reason, incomplete activation of botulinum neurotoxins may further contribute to their immunogenicity (Aoki and Guyer 2001). To become activated, botulinum neurotoxins must be nicked (i.e., cleaved by a protease), which produces two polypeptide fragments (a ~100-kDa heavy chain and a 50-kDa light chain) that remain tethered together by a disulfide bond (Aoki and Guyer 2001). Based on the literature, BoNT/A is approximately 95 % nicked (and therefore activated) by an endogenous bacterial protease before it is released from the cell (Das Gupta and Suathyamoorthy 1984). In contrast, much lower levels of BoNT/B are endogenously nicked, so the toxin must be exposed to proteases during the manufacturing process to produce activation (Das Gupta and Sugiyama 1976; Moyer and Setler 1994; Setler 2002). Despite this, it is reported that approximately 25–30 % of the BoNT/B product rimabotulinumtoxinB remains inactive (Callaway 2004), which in part may explain the high rates of reported immunogenicity with BoNT/B (Jankovic et al. 2006; Dressler and Bigalke 2005; MYOBLOC® [package insert] 2010).

Botulinum neurotoxins may become inactivated during the manufacturing process, especially if conditions cause aggregation and/or oxidation as mentioned above. In addition, toxins may degrade if suboptimally stored between the time of manufacture and clinical use, which may increase the amount of inactive toxin in, and the immunogenicity of, a therapeutic product (Hunt and Clarke 2009).

#### Antigenic protein load

Only the 150-kDa core BoNT is capable of stimulating the formation of neutralizing antibodies, so when the relative immunogenic potential of a botulinum neurotoxin product is calculated, only the mass of the 150-kDa core BoNT component should be considered. This may be referred to as the “antigenic protein load” and is different from overall neurotoxin protein amount, which includes both the core neurotoxin and NAPs. For onabotulinumtoxinA, which consists of neurotoxin complexes that are ~900-kDa (Lietzow et al. 2008), the 150-kDa core BoNT component is only approximately one-sixth of the total mass. Therefore, a 100 U vial of onabotulinumtoxinA, which contains approximately 5 ng of neurotoxin complex, would be expected to have an antigenic protein load of ~0.8 ng/vial (Table 1). IncobotulinumtoxinA, which contains only the 150-kDa core BoNT without NAPs, has an antigenic protein load of 0.44–0.6 ng/vial (Roggenkamper et al. 2006; Frevert and Dressler 2010). The antigenic protein load of abobotulinumtoxinA is unknown, as the overall size of the neurotoxin complex is unknown, but the total neurotoxin complex protein load is reported as 4.35 ng/500 U vial (Pickett et al. 2007). One 5,000 U vial of rimabotulinumtoxinB consists of approximately 50 ng neurotoxin complex that is ~700-kDa. This equates to an antigenic protein load of ~10.7 ng/5,000 U vial (Callaway 2004; Setler 2000).

**Table 1:** Protein amounts in different botulinum neurotoxin products

Botulinum neurotoxin products	Total protein (ng/vial)	Antigenic protein load (ng/vial)
AbobotulinumtoxinA (500 U vial)	~5	Unknown
IncobotulinumtoxinA (100 U vial)	~0.6	~0.6
OnabotulinumtoxinA (100 U vial)	~5	~0.8
RimabotulinumtoxinB (5,000 U vial)	~50	~10.7

Another measure that has been used by some authors to assess the relative antigenicity of different botulinum neurotoxins is “specific biologic activity” (SBA) (Wohlfarth et al. 1997; Dressler and Hallett 2006). For botulinum neurotoxin products, SBA has been defined as the ratio between the units of a botulinum neurotoxin product in a vial (representing the potency of a particular product in the mouse lethality assay) and the mass of neurotoxin in the vial. Given that only the 150-kDa core BoNT can stimulate neutralizing antibody formation, it would seem more appropriate for SBA to be calculated based solely on the antigenic protein load per vial. For example, the SBA for onabotulinumtoxinA would be 120 U/ng based on a 100 U vial and a 150-kDa mass of 0.83 ng/vial. However, SBA is based on labeled unit values and comparison presupposes direct correlation of unit values from product to product, which is specifically prohibited (as stated in the product regulatory labels of commercially available botulinum neurotoxin products worldwide); therefore, comparisons of SBA among botulinum neurotoxin products are not valid.

#### Accessory proteins and excipients

All of the currently available botulinum neurotoxin formulations, with the exception of incobotulinumtoxinA, include NAPs. Patients may develop antibodies against NAPs, but by definition, these antibodies are non-neutralizing (Göschel et al. 1997; Joshi et al. 2011). For example, in a study by Joshi et al. (2011) mice immunized against the core neurotoxin showed no decrease in locomotor activity after BoNT/A injection, whereas BoNT/A was still able to depress activity in mice immunized against any of three hemagglutinin NAPs, indicating that antibodies against the NAPs do not interfere with the function of the core neurotoxin. Although two pre-clinical studies have suggested that NAPs may act as immunologic adjuvants to increase the antigenicity of BoNT (Lee et al. 2005; Kukreja et al. 2009), appropriate caution is warranted when interpreting their results and their implications for clinical practice because the methodology used may have increased immunogenicity in several ways (Atassi 2006). Both studies used formaldehyde-treated proteins (a process known to enhance immunogenicity) and administered higher concentrations of botulinum neurotoxin than those used clinically and in more frequent doses, which would be expected to significantly enhance immunogenicity (Lee et al. 2005; Kukreja et al. 2009). Furthermore, both studies used non-commercially produced botulinum neurotoxin preparations for which the purity is unknown, and an adjuvant was co-administered with the toxin in the one study (Kukreja et al. 2009). These studies were also performed in mice and rabbits and thus results cannot be extrapolated to humans.

A pre-clinical study of BoNT antibody formation after vaccination with abobotulinumtoxinA, incobotulinumtoxinA, or onabotulinumtoxinA found higher rates of neutralizing antibody formation for abobotulinumtoxinA versus onabotulinumtoxinA and incobotulinumtoxinA (Blümel et al. 2006). Since abobotulinumtoxinA has fewer NAPs compared with onabotulinumtoxinA, yet a higher reported rate of neutralizing antibody formation, this study may suggest that NAPs do not contribute to antibody formation and that other factors may influence the immunogenic profile of botulinum neurotoxin products. One possible factor could be flagellin, which was recently identified as a protein component of the abobotulinumtoxinA bulk toxin (Panjwani et al. 2008). Flagellin is a constituent protein of the bacterial locomotor apparatus that interacts with the Toll-Like Receptor 5 (TLR5) initiating an innate immune response (Yoon et al. 2012). Flagellin is known to be an immunologic adjuvant (Mizel and Bates 2010).

To date, there are no published clinical data to support the hypothesis that NAPs can increase the immune response to botulinum neurotoxin products. In fact, it has been proposed that NAPs cover and sterically restrict access to the BoNT/A site at which most neutralizing antibodies form (Chen et al. 1997; Gu et al. 2012) and may thus potentially reduce the immunogenicity of botulinum neurotoxin products that are shielded by NAPs.

All commercially available botulinum neurotoxin products contain albumin, an excipient that is added to stabilize the product and to aid in the recovery of the neurotoxin from the vial (Bigalke et al. 2001; Schantz and Johnson 1992). The albumin is derived from human sources and is reported to be generally non-immunogenic, with a 0.011 % incidence of anaphylactic responses to infusion at high concentrations (Ring and Messmer 1977). Thus, it is unlikely to induce a significant immune response, especially at the low doses in which it is used in formulating botulinum neurotoxin products (Bosse et al. 2005). Other excipients used in botulinum neurotoxin formulations include small sugars (sucrose, lactose) and salts (sodium chloride, sodium succinate) (DYSPORT™ [package insert] 2010; XEOMIN® [package insert] 2011; MYOBLOC® [package insert] 2010), which are unlikely to induce or enhance an immune response.

### Treatment-related factors

#### Dose

Reports that the immune response to an antigen is generally dose-dependent (Göschel et al. 1997; Aoki and Guyer 2001; Atassi 2004) have led to the hypothesis that botulinum neurotoxin immunogenicity may be related to the dose that is injected. Indeed, the balance of currently available evidence in the published literature suggests that the development of neutralizing antibodies to BoNT is positively correlated with the cumulative dose. In an early retrospective study, patients with neutralizing antibodies to pre-1997 onabotulinumtoxinA were found to have received a higher total cumulative dose (mean 1,709 ± 638 U over 2.5 years) than patients without neutralizing antibodies (mean 1,066 ± 938 U over 2.4 years; *P* < 0.01) (Jankovic and Schwartz 1995). In another study, patients with CD who developed resistance to onabotulinumtoxinA had received higher doses per treatment than non-resistant patients (Greene et al. 1994). A higher cumulative dose and/or mean dose per treatment have been associated with neutralizing antibodies against type A botulinum toxin products and secondary non-response in two other studies (Lange et al. 2009; Dressler and Dimberger 2000), and lower rates of neutralizing antibodies against BoNT have been reported in patients with conditions requiring lower BoNT/A doses (e.g., blepharospasm, hemifacial spasm, or cosmetic use) compared with higher-dose applications (e.g., focal spasticity or torticollis) (Lange et al. 2009). One study has reported that patients who develop neutralizing antibodies require higher and more frequent doses to maintain comparable levels of treatment effectiveness (tachyphylaxis) (Dressler et al. 2002), so if doses and dosing intervals remain consistent, this may indirectly suggest that patients had not developed neutralizing antibodies. In fact, a retrospective review of the use of onabotulinumtoxinA in patients with CD (*N* = 172) over a 2-year period found that the mean doses of and intervals between onabotulinumtoxinA injections were consistent (Brashear et al. 2005). It is important to note, however, that this study (Brashear et al. 2005) was not designed to evaluate immunogenicity and would have been underpowered to detect a small effect in the range currently estimated for antibody formation (~1 % or less) (Brin et al. 2008).

#### Treatment intervals

Immunogenicity may be related to the frequency of injection. In the early days of botulinum neurotoxin therapy for CD, “booster” injections were often given to patients 2–3 weeks after the initial dose if the first dose had been deemed not to have produced an adequate response. Two studies found that patients who developed secondary non-response to onabotulinumtoxinA received more frequent injections and/or had more booster injections than patients who did not develop resistance (Greene et al. 1994; Dressler et al. 2002). In addition, a recent evaluation of serum samples from secondary non-responders to both abobotulinumtoxinA and pre-1997 onabotulinumtoxinA revealed that higher proportions of patients with treatment intervals of 1–2 months tested positive for neutralizing antibodies compared with those with treatment intervals of 4–13 months (Lange et al. 2009). These results suggest that shorter botulinum neurotoxin injection intervals (i.e., <2 months apart) may increase the risk for neutralizing antibody formation and treatment non-response. As a result, longer injection intervals (based on the expected duration of clinical effect and to lower the risk for neutralizing antibody formation) have been adopted as standard clinical practice and are reflected in recommended treatment schedules for all botulinum neurotoxin (as indicated in US product labels) (DYSPORT™ [package insert] 2010; XEOMIN® [package insert] 2011; MYOBLOC® [package insert] 2010).

#### Previous exposure or vaccinations

Prior vaccination or toxin exposure may also affect immunogenicity. Many individuals who received vaccination against botulinum toxin (e.g., US military personnel) appear to retain antibody titers and would not be expected to respond to botulinum neurotoxin therapeutic treatments (Smith and Rusnak 2007; Hatheway and Dang 1994). Likewise, survivors of past botulism exposure may have generated antibodies against BoNT and may not respond to therapeutic botulinum neurotoxin injection (Hatheway and Dang 1994).

Previous exposure to botulinum neurotoxin products may influence the immune response to BoNT. Patients who develop secondary non-response to one BoNT serotype are often switched to the other available serotype, but some studies suggest that the re-establishment of therapeutic efficacy is transient and incomplete (Dressler et al. 2003; Factor et al. 2005; Dressler and Eleopra 2006). One anecdotal report has suggested that secondary non-response to BoNT/A may be overcome with another BoNT/A formulation (Badarny et al. 2008); however, this strategy would seem unlikely, as BoNT serotypes are defined immunologically.

As the tetanus toxin and BoNTs A and B show >50 % amino acid similarity (Whelan et al. 1992; Hutson et al. 1994), and anti-tetanus toxin antibodies have been shown to bind to BoNTs A and B in vitro (Halpern et al. 1989; Dolimbek et al. 2002), it has been theorized that prior immunization against tetanus may prime a patient’s immune system to BoNT (Dolimbek et al. 2002). A pre-clinical study conducted in mice showed that the presence of prior active immunity against tetanus toxins did not enhance the host antibody response against injected BoNT (Dolimbek et al. 2002); however, no clinical studies have been performed to examine whether this holds true for humans.

## Laboratory and clinical tests for detection of anti-BoNT antibodies

### In vitro assays

In vitro analyses using enzyme-linked immunosorbent assays (ELISA), Western blots, and radioimmunoprecipitation assays (RIPA) can provide quantitative estimates of the binding antibody titer against the core neurotoxin. Although the core BoNT is used as the capture antigen, these assays are not capable of distinguishing between neutralizing and non-neutralizing antibodies (Hatheway and Dang 1994). In other words, these assays detect antibodies that bind to the core BoNT, including those that may or may not be neutralizing, and thus would not necessarily lead to reduced efficacy. Although these assays are sensitive, they are less specific than bioassays (described below) and results often do not correlate well with in vivo or clinical test results (Hatheway and Dang 1994; Hanna and Jankovic 1998; Hanna et al. 1999; Lawrence and Moy 2009). Use of these assays exclusively to identify the presence of neutralizing antibodies is therefore not appropriate. For this reason, these assays are used only as the first step of a clinical immunogenicity screening strategy and are followed by a second assay (usually functional bioassay) to confirm the presence of neutralizing antibodies.

### Bioassays

As described above, only neutralizing antibodies can inhibit the biological activity of the toxin and potentially lead to treatment failure. Thus, it is important to use assays that can identify the presence of neutralizing antibodies. Bioassays include the mouse protection assay (MPA) and the mouse diaphragm assay (MDA), both of which have the benefit of distinguishing between neutralizing and non-neutralizing antibodies. The MPA, which tests the ability of the patient serum to protect mice from the effects of intraperitoneal (i.p.) administered lethal doses of BoNT, is considered to be the standard method for detection of neutralizing antibodies (Hatheway and Dang 1994). In the MPA, patient serum is incubated with a known dose of the toxin and the serum/neurotoxin mix is injected into mice. Mouse survival indicates the presence of neutralizing antibodies in the serum and antibody levels can be quantified by comparison with simultaneously tested standard anti-toxins (Hatheway and Dang 1994). An early study investigated the correlation between MPA and clinical responsiveness to treatment, and found a very high specificity (100 %, no false positives; no patients who were MPA positive had a clinical response) but lower sensitivity (~47 %; higher false negative rate; patients with clinical non-response were not positive on the MPA), perhaps because neutralizing antibody levels are below the limit of detection (Hanna and Jankovic 1998). However this finding does not appear to be universal as some patients with the confirmed presence of neutralizing antibodies by MPA continue to respond to treatment (Naumann et al. 2009; Carruthers et al. 2004). This may be a result of fluctuations in testing conditions or patient serum titers as well as variations among different laboratories. In addition, the MPA has several other limitations, including use of laboratory animals, expense, and length of time to obtain results, and the results are semiquantitative (Hanna et al. 1999; Dressler et al. 2000).

For the MDA, patient serum is mixed with standardized neurotoxin doses and the combination is applied to an excised mouse phrenic nerve, and the half of the diaphragm that it innervates in a solution that maintains the physiologic condition of the muscle as if it were intact (Göschel et al. 1997). The amount of antibody present in the serum is determined using a calibration curve of the time required to decrease diaphragm contraction by 50 % (Göschel et al. 1997). The detection limit of the MDA was originally reported as 0.3 mU/ml (Göschel et al. 1997). However, a recent comparison of the MDA and MPA found that the detection limit of the MDA was 0.17 versus 1 mU/ml for the MPA, which would indicate that the sensitivity of the MDA is about six-fold higher than that of the MPA (Fink et al. 2009). Although the MDA is more sensitive, it may be too sensitive to predict secondary non-response and may yield a high false-positive rate if appropriate antibody titer thresholds are not employed.

A combination of in vitro and bioassays has been used to detect neutralizing antibodies. In vitro assays (i.e., ELISAs or RIPAs), which are sensitive but do not distinguish between neutralizing and non-neutralizing antibodies, are first used to detect the presence of any BoNT antibodies. Samples found to be positive are then screened using the more specific MPA or MDA to detect neutralizing antibodies (Kanovsky et al. 2009; Lawrence and Moy 2009; Truong et al. 2010). It is currently unclear whether the two-step process improves the clinical utility of the results of neutralizing antibody testing.

### Clinical assays

Several clinical tests (including the frontalis antibody test [FTAT], unilateral brow injection [UBI], and extensor digitorum brevis [EDB] assay) may be used to evaluate a patient’s sensitivity to botulinum neurotoxin, which, if diminished, may suggest the presence of neutralizing antibodies. In the FTAT and UBI, a low test dose of BoNT is injected unilaterally into a patient’s frontalis or corrugator muscle, respectively (Hanna and Jankovic 1998; Brin et al. 2008). Patients who exhibit symmetry of forehead wrinkling or glabellar furrowing following the test botulinum neurotoxin injection are deemed insensitive to botulinum neurotoxin, which may be mediated by neutralizing antibodies (Hanna and Jankovic 1998). The primary advantage of clinical tests is that, in contrast to laboratory tests, they provide clear evidence of the presence or absence of clinical responsiveness to botulinum neurotoxin, so their results may be of greater utility to help guide clinical decisions regarding future botulinum neurotoxin treatments. These tests are easy to use, have low rates of false positivity for secondary non-response, and are relatively inexpensive compared with the mouse assays (Hanna and Jankovic 1998; Hanna et al. 1999). The FTAT and UBI correlate well with the in vitro assays and bioassays, with specificities ranging from 81 to 100 % (i.e., a low false positive rate) with the RIPA and Western blot assays, and 100 % with the MPA (Hanna and Jankovic 1998; Hanna et al. 1999). Sensitivities of these in vitro assays varied when correlated with clinical tests, ranging from 30 to 90 %. However, the clinical tests do not directly measure the presence of BoNT-neutralizing antibodies.

In the EDB assay, botulinum neurotoxin is injected into the EDB muscle of patients suspected of having secondary non-response as a result of the presence of neutralizing antibodies (Kessler and Benecke 1997). Compound muscle action potentials (CMAPs) are measured electrophysiologically and pre- and post-injection changes in CMAP amplitudes are compared. Although the assay is carried out in individual patients, which is beneficial, the results are non-quantitative and the designation of a “positive” result is somewhat subjective. In one study, EDB assay results appear to correlate well with those from the MPA, since patients with neutralizing antibodies showed no decrease in CMAP amplitude (Kessler and Benecke 1997). However, another study showed little correlation between the EDB and MDA but did show a significant difference in CMAP amplitudes between healthy controls and secondary non-responders (Garcia et al. 2009).

## Clinical immunogenicity of BoNT products

As mentioned above, all therapeutic proteins have the potential to be immunogenic and can lead to neutralizing antibody formation. However, as compared with most protein biologics, the amount of protein injected during treatment with any of the currently approved type A botulinum neurotoxins is extremely low (Table 2) and may account for the relatively low rate of antibody-induced treatment failure observed with BoNT/A products. Furthermore, a meta-analysis of data of seroconversion rates from pivotal trials of onabotulinumtoxinA in several clinical indications did not find an association between a change in antibody status and other local or systemic immune-related responses (Naumann et al. 2010).

**Table 2:** Examples of dosing and frequency of administration of commonly used biologics

Non-proprietary name	Trade name (Reference)	Indication	Dose	Frequency
OnabotulinumtoxinA	BOTOX® (BOTOX® [package insert] 2011)	Cervical dystonia	236 U (1.95 ng 150 kD neurotoxin)	Every 3–4 months (BOTOX® [package insert] 2011)
	BOTOX® Cosmetic (BOTOX® Cosmetic [package insert] 2011)	Glabellar lines	20 U (0.17 ng 150 kD neurotoxin)	Every 4 months
Adalimumab	Humira® (HUMIRA® [package insert] 2011)	Rheumatoid arthritis, plaque psoriasis, ankylosing spondylitis, Crohn’s disease	40 mg (maintenance dose)	Every other week (HUMIRA® [package insert] 2011)
Interferon β-1a	Avonex® (AVONEX® [package insert] 2006)	Multiple sclerosis	30 μg (AVONEX® [package insert] 2006)	Weekly
	Rebif® (Rebif® [package insert] 2005)	Multiple sclerosis	22 or 44 μg	Three times per week (Rebif® [package insert] 2005)
	Extavia® (Extavia® [package insert] 2009)	Multiple sclerosis	0.25 mg (Extavia® [package insert] 2009)	Every other day

The following section will review the clinical immunogenicity data for the four commercially available botulinum neurotoxin products in various indications (Table 3). However, it is important to note that neutralizing antibody rates cannot be directly compared among products because of differences in antibody tests and variations in patient samples (including sample handling and timing of collection), disease states, medication usage, and variations in the time period of follow-up.

**Table 3:** Frequency of neutralizing antibodies to different botulinum neurotoxin formulations

Indication	AbobotulinumtoxinA	IncobotulinumtoxinA	OnabotulinumtoxinA^a^	RimabotulinumtoxinB
Cervical dystonia	1–3 % (DYSPORT™ [package insert] 2010; Göschel et al. 1997)	0–1 %^b^ (2010; Benecke 2009)	0–1 % (BOTOX® [package insert] 2011; Jankovic et al. 2003)	10–44 % (MYOBLOC® [package insert] 2010; Jankovic et al. 2006; Dressler and Bigalke 2005)
Blepharospasm/facial movement disorder	NA	NA	NA	NA
Spasticity (upper limb)	0^c^ (Bakheit et al. 2004)	0^d^ (Kanovsky et al. 2009)	0.5 % (BOTOX® [package insert] 2011; Elovic et al. 2008; Yablon et al. 2007)	0^e^ (Brashear et al. 2004)
Hyperhidrosis	NA	NA	0.2 % (BOTOX® [package insert] 2011)	NA
Chronic migraine	NA	NA	0 (BOTOX® [package insert] 2011)	NA
Urinary incontinence due to neurogenic detrusor overactivity	NA	NA	0 (Cruz et al. 2011)	NA
Cosmetic	0 (DYSPORT™ [package insert] 2010; Monheit and Cohen 2009; Moy et al. 2009)	0 (Imhof and Kühne 2011)	0 (Carruthers et al. 2004; Kawashima and Harii 2009)	NA

*NA* not available

^a^Current formulation

^b^Based on one study that included patients with CD and blepharospasm and one open-label study of 100 patients with CD

^c^Based on one small study with 41 patients

^d^Based on one study with 73 patients

^e^Based on one small study with 10 rimabotulinumtoxinB-treated patients

## Cervical dystonia

All four commercially available botulinum neurotoxin products are approved for the treatment of CD in the United States.

### OnabotulinumtoxinA

The immunogenicity rate of the current formulation of onabotulinumtoxinA in patients with CD as reported in the product label is 1.2 % (BOTOX® [package insert] 2011). These data are from an open-label, long-term, observational study in 326 botulinum neurotoxin-naïve patients with CD who received a median of nine onabotulinumtoxinA treatments over a mean of 2.5 years (Brin et al. 2008). Four patients (1.2 %) had positive MPA tests during the course of up to 15 treatment cycles, all of whom were responders at the time of MPA testing, but 3 eventually lost responsiveness. Of these four, the one clinically responsive patient and one of the non-responsive patients were responsive on the FTAT or UBI tests; one of the other clinically non-responsive patients was non-responsive on the FTAT/UBI, and the other patient withdrew before further testing. These results are consistent with those reported by Jankovic et al. from a prospective study that compared the immunogenicity rate of the current onabotulinumtoxinA formulation with that of the original version in 249 patients with CD (Jankovic et al. 2003). The incidence of neutralizing antibodies as determined by MPA was 0 % (0/119) for current onabotulinumtoxinA alone versus 9.5 % (4/42) for original onabotulinumtoxinA (*P* < 0.01). After adjustment for covariate effects of age and cumulative dose, the risk of developing antibody formation with the current formulation of onabotulinumtoxinA was reduced by a factor of six compared with the original version.

The immunogenicity rate of the original formulation of onabotulinumtoxinA in patients with CD was examined in several long-term studies. Mejia et al. (2005) conducted a longitudinal follow-up study of 45 patients with various movement disorders treated for >12 years. The first injections were performed from 1985 to 1989 and, as a result, it can be assumed that all patients initially received the original onabotulinumtoxinA formulation. The dose per treatment session was increased at the last visit versus the first visit (*P* < 0.0001). Antibody testing was performed for 22 patients exhibiting a less than satisfactory response on two consecutive visits but only 4 (8.9 %) were MPA-positive (16 MPA-negative patients resumed responsiveness after dose adjustments; 2 persisted as non-responders). Hsiung et al. (2002) retrospectively analyzed 106 patients with CD who were treated with original onabotulinumtoxinA for 10 years and saw a trend toward dose increases over time, but this was not considered significant. The incidence of secondary resistance was 1.7 cases/100 person-years of observation, but no serum antibody tests were performed; therefore, it was not possible to determine whether the dose increase was associated with neutralizing antibody formation.

### AbobotulinumtoxinA

As reported in the prescribing information, ~3 % of patients with CD treated with abobotulinumtoxinA developed binding or neutralizing antibodies (DYSPORT™ [package insert] 2010). In the published literature, the immunogenicity rate in patients with CD or focal dystonia treated with abobotulinumtoxinA for up to 2 years ranges from 0 to 3.1 % using MPA or ELISA (Moore and Blumhardt 1991; Anderson et al. 1992; Zuber et al. 1993; Brans et al. 1995). Göschel et al. (1997) tested 150 patients with CD who had received abobotulinumtoxinA and found 1 non-responding patient (0.7 %) who tested positive for neutralizing antibodies via the MDA. Truong et al. (2010) conducted a randomized, double-blind, placebo-controlled study of 116 patients with CD treated with 500 U abobotulinumtoxinA or placebo, with an open-label extension of four treatment cycles. Blood samples were first screened by RIPA and positive samples were tested for neutralizing antibodies via the MPA. One patient (0.9 %) developed neutralizing antibodies by the end of the study but was a clinical responder during the double-blind and open-label trial phases. A similarly designed study with double-blind and open-label phases showed that 3/136 (2.2 %) abobotulinumtoxinA-treated patients developed neutralizing antibodies as measured by the MPA (Coleman et al. 2010).

The immunogenicity rate of abobotulinumtoxinA in patients with CD was examined in two long-term, open-label studies. One study tested 303 patients with CD who had been treated with ≥6 injections of abobotulinumtoxinA. Neutralizing antibodies were detected by MPA, MDA, or EDB in 9 of 17 secondary non-responders (Kessler et al. 1999). The authors used a reference group consisting of patients who received ≥6 injections (303 patients who were still receiving therapy plus 54 patients who discontinued the study) and determined that the antibody frequency was 2.5 % (9/357 patients). Secondary non-responders who tested positive differed significantly from responders in that they received higher doses per session, had shorter treatment intervals, and had higher numbers of booster sessions. In the other long-term, open-label study of abobotulinumtoxinA in patients with CD, 3 of 90 patients (3.3 %) treated with abobotulinumtoxinA for 10 to 12 years were secondary non-responders (Haussermann et al. 2004). Testing with the in vitro MDA failed to find evidence of antibodies in these patients.

### IncobotulinumtoxinA

In the incobotulinumtoxinA development program (which consisted of studies in patients with CD, blepharospasm, and upper limb spasticity), 1.1 % of patients (12/1,080 patients) treated with incobotulinumtoxinA developed neutralizing antibodies during the course of their study as measured via ELISA followed by MDA (FDA Approval Package for Xeomin® 2010). In a placebo-controlled study of incobotulinumtoxinA (120 U or 240 U) in 233 patients with CD, 4 patients developed positive antibody tests during the placebo-controlled phase of the trial and 4 other patients developed antibodies during open-label treatment for an overall rate of 3.4 % (8/233 patients) (FDA Approval Package for Xeomin® 2010). It should be noted that the majority of patients in these studies had been previously treated with other botulinum neurotoxins; however, patients who seroconverted after receiving incobotulinumtoxinA did not demonstrate the presence of neutralizing antibodies before enrollment. An open-label study of 100 patients with CD, half of whom had previously been treated with onabotulinumtoxinA, abobotulinumtoxinA, or rimabotulinumtoxinB, showed that no patients tested positive for neutralizing antibodies via the MDA after continuous treatment with incobotulinumtoxinA for up to 2 years (Benecke 2009).

### RimabotulinumtoxinB

The rimabotulinumtoxinB prescribing information states that the immunogenicity rates in patients with CD (*N* = 446), based on ELISA, were 12, 20, 36, and 50 % at baseline and after 6, 12, and 18 months of rimabotulinumtoxinB treatment, respectively (MYOBLOC® [package insert] 2010). Neutralizing antibodies were generally not detected until after 6 months of treatment and estimated rates of neutralizing antibodies were 10 % at 12 months and 18 % at 18 months based on the MDA (MYOBLOC® [package insert] 2010).

Jankovic et al. (2006) conducted a 42-month observational study of rimabotulinumtoxinB in 100 patients with CD who may have received prior treatment with botulinum neurotoxin type A products, type B products, or both. The proportion of patients with BoNT/A-neutralizing antibodies assessed by MPA decreased from 13.0 % at baseline to 2.5 % over the course of the study, whereas 34.4 % developed de novo immunoresistance to BoNT/B. The development of BoNT/B antibodies was reported to correlate with total cumulative dose but not with prior exposure to botulinum neurotoxin type A or B products. A small (*N* = 9) study of de novo therapy for CD with rimabotulinumtoxinB showed that 44 % (4/9) of patients developed secondary resistance and those four patients had high titers of antibodies against BoNT/B as shown by the MDA (Dressler and Bigalke 2005).

Four long-term studies were carried out to determine the long-term immunogenicity of BoNT/B. Patients with CD (*N* = 1159) received 2,500–25,000 U rimabotulinumtoxinB approximately every 12 weeks, and antigenicity rates, as measured by MDA, were 33.0, 42.0 to 44.0, and 38.6 % over the 2-, 4-, and 7-year studies, respectively (Birmingham et al. 2010; Chinnapongse et al. 2010; Lew et al. 2010; Reinhard et al. 2010). Despite these relatively high rates, ≥73 % of the antibody-positive patients remained in each trial for over 2 years, and efficacy analyses showed no difference between mouse-neutralizing antibody positive and negative patients. Furthermore, of those who discontinued as a result of perceived lack of effect, the majority were antibody negative. The authors thus suggest that the development of neutralizing antibodies does not correlate with loss of effect.

## Blepharospasm/facial movement disorders

### OnabotulinumtoxinA

OnabotulinumtoxinA is indicated for the treatment of blepharospasm associated with dystonia. An early study of the pre-1997 formulation of onabotulinumtoxinA (*N* = 42) for the treatment of blepharospasm, hemifacial spasm, and CD found antibodies in 57 % (24/42) of patients (Siatkowski et al. 1993). Of note, this study used a sphere-linked immunodiagnostic assay to detect antibodies, which does not discriminate between neutralizing and non-neutralizing antibodies. The presence of antibodies did not appear to affect the response to treatment, which suggests that the antibodies were non-neutralizing or the titers were too low to exhibit a clinical effect.

Consistent with other reports suggesting that larger doses lead to antibody formation, another study of the original formulation of onabotulinumtoxinA in patients with ocular movement disorders found that 4 % (2/45) of patients who received <500 U/year of pre-1997 onabotulinumtoxinA (Oculinum) had positive MPA, whereas 63 % of patients (27/43) who received ≥500 U/year had positive MPA results (Hatheway and Dang 1994). These data suggest that the incidence of neutralizing antibody development to the pre-1997 formulation of onabotulinumtoxinA increased with the cumulative dose, although no clinical correlation was provided.

### AbobotulinumtoxinA

No data on the immunogenicity of abobotulinumtoxinA in patients with blepharospasm or facial movement disorders are available from clinical trials. Lange et al. (2009) evaluated neutralizing antibody levels in serum samples collected from 1995 to 2000 from 503 patients classified clinically as secondary non-responders to abobotulinumtoxinA or onabotulinumtoxinA. Overall, fewer than half of the patients in the study, all of whom were secondary non-responders, tested positive for neutralizing antibodies, indicating that factors other than antibody development contributed to treatment failure. This may also point to the limitations of using a mouse assay to predict the neutralization capability of antibodies in the complex human system. Furthermore, of the patients with blepharospasm, 4/21 patients (19 %) treated with abobotulinumtoxinA and 1/7 patients (14 %) treated with onabotulinumtoxinA were positive for neutralizing antibodies. Given the time frame of the sampling, it is likely that many of the patients treated with onabotulinumtoxinA received the pre-1997 product.

### IncobotulinumtoxinA

Data on the immunogenicity of incobotulinumtoxinA from two Phase III clinical trials (≥35 U or ≤50 U per eye) in patients with blepharospasm, as measured by ELISA followed by MDA, were submitted to the US Food and Drug Administration (FDA Approval Package for Xeomin® 2010). In these trials, 2/222 patients (1 %) showed neutralizing antibodies at baseline and 1 patient developed de novo antibodies that were not present at baseline. All patients in this trial had been previously treated and had reported a satisfactory clinical response to onabotulinumtoxinA.

### RimabotulinumtoxinB

No information is available regarding the antigenicity rate of rimabotulinumtoxinB in the treatment of facial movement disorders.

## Adult spasticity (upper limb)

### OnabotulinumtoxinA

OnabotulinumtoxinA is approved in the United States for the treatment of upper limb spasticity, and the product information states that 2/380 patients (0.5 %) developed neutralizing antibodies during treatment for this indication (BOTOX® [package insert] 2011). These results are consistent with those from a pooled analysis by Yablon et al. (2007), which examined antibody development in 191 post-stroke spasticity patients from three studies (one of which was also included in the US prescribing information) who received at least one onabotulinumtoxinA injection (100–400 U) over 12–42 weeks of treatment (Brashear et al. 2002; Gordon et al. 2004; Turkel et al. 2002). Neutralizing antibodies to onabotulinumtoxinA were detected by MPA in 1/191 (0.5 %) patients with available serum samples. This patient did not have an analyzable baseline serum sample and did not respond to onabotulinumtoxinA at any time during the study, and is one of the 2 patients cited in the onabotulinumtoxinA label. A long-term, open-label study of 279 patients with post-stroke upper limb spasticity who received up to five intramuscular injections of onabotulinumtoxinA (200–400 U) found neutralizing antibodies by MPA in 1/224 patients (0.45 %) with serum samples (Elovic et al. 2008). This patient had diminished responses with the final treatments and clinical non-response was confirmed by FTAT. In this study, 76 % of patients received a starting dose of ≥250 U with an injection frequency of not less than every 12 weeks.

### AbobotulinumtoxinA

The only published study of abobotulinumtoxinA in post-stroke upper limb spasticity is an open-label trial of 41 patients injected with 1,000 U of abobotulinumtoxinA for three treatment cycles. No neutralizing antibodies were detected by MPA (Bakheit et al. 2004).

### IncobotulinumtoxinA

Kanovsky et al. (2009) conducted a randomized, double-blind, placebo-controlled trial in 73 patients exposed to a single cycle of incobotulinumtoxinA (median 320 U; range 80–435 U). No neutralizing antibodies were detected by the two-step process of non-specific fluorescence immunoassay followed by MDA.

### RimabotulinumtoxinB

A 16-week, randomized, double-blind, placebo-controlled study in 10 patients with upper limb spasticity treated with 10,000 U of rimabotulinumtoxinB followed by a 12-week open-label extension found no neutralizing antibodies detected by MPA (Brashear et al. 2004).

## Hyperhidrosis

OnabotulinumtoxinA is approved in the United States for treatment of hyperhidrosis. In the pivotal studies, 1 in 445 patients (0.2 %) with primary axillary hyperhidrosis developed neutralizing antibodies following onabotulinumtoxinA treatment as measured via MPA (BOTOX® [package insert] 2011). A further analysis of seroconversion in hyperhidrosis pivotal trials identified 4/871 patients (0.5 %) who became positive for neutralizing antibodies through the course of the trials, although all 4 maintained clinical responsiveness (Naumann et al. 2010). No information is available on the immunogenicity rates of abobotulinumtoxinA, incobotulinumtoxinA, or rimabotulinumtoxinB in the treatment of hyperhidrosis.

## Chronic migraine

OnabotulinumtoxinA is indicated for treatment of chronic migraine. In the pivotal studies, 0/406 migraine patients (0 %) developed neutralizing antibodies following treatment with onabotulinumtoxinA after 24 weeks (two treatment cycles) (BOTOX® [package insert] 2011). Antigenicity rates were not reported in a small, open-label trial of abobotulinumtoxinA for the treatment of chronic migraine (Menezes et al. 2007), and neither incobotulinumtoxinA nor rimabotulinumtoxinB have been studied for this indication.

## Urinary incontinence due to neurogenic detrusor overactivity

OnabotulinumtoxinA is also indicated for treatment of urinary incontinence due to detrusor overactivity in patients with a neurologic condition (i.e., multiple sclerosis or spinal cord injury). No neutralizing antibodies were detected after 12 weeks of treatment in 180 patients receiving either 200 or 300 U of onabotulinumtoxinA in a pivotal Phase III trial in this condition (Cruz et al. 2011). Antigenicity rates have not been reported in the trials of abobotulinumtoxinA for the treatment of urinary incontinence that have been conducted to date (Ehren et al. 2007; Grise et al. 2010; Grosse et al. 2009); incobotulinumtoxinA and rimabotulinumtoxinB have not been studied for this indication.

## Cosmetic applications

### Onabotulinumtoxin

OnabotulinumtoxinA dosing for facial aesthetics is generally lower and less frequent than for therapeutic indications (BOTOX® Cosmetic [package insert] 2011; BOTOX® [package insert] 2011). An analysis of data from two identical randomized, double-blind, placebo-controlled studies of onabotulinumtoxinA cosmetic (20 U) in patients with glabellar lines showed that none of the 159 evaluable patient samples (including 3 that were positive at baseline) were MPA positive for neutralizing antibodies at day 120 (Carruthers et al. 2004). Four out of 283 patients (1.4 %) tested positive at one or more time points between pre-treatment and post-treatment but all were considered responders. Similarly, a 64-week, open-label trial in which botulinum neurotoxin-naïve patients with glabellar lines received four injection cycles of 10 or 20 U onabotulinumtoxinA cosmetic found that none of the 363 patients enrolled tested positive for neutralizing antibodies at any point during the study (Kawashima and Harii 2009).

### AbobotulinumtoxinA

The abobotulinumtoxinA prescribing information (DYSPORT™ [package insert] 2010) reports an analysis of the Phase III trials of 1,554 subjects who had up to nine cycles of treatment of 250 U for glabellar lines. Two subjects (0.13 %) tested positive for binding antibodies at baseline by initial screening via RIPA and 3 more tested positive after receiving treatment; none were positive for neutralizing antibodies by the MPA nor did any of these patients show reduced efficacy.

Two of the five published studies in the United States supporting clinical development of abobotulinumtoxinA for glabellar lines reported on immunogenicity (Monheit and Cohen 2009; Moy et al. 2009). None of the patients (*N* = 1968) who received ≤6 injections of 50 U of abobotulinumtoxinA over a 13- to 17-month period developed antibodies.

### IncobotulinumtoxinA

An open-label Phase III trial of incobotulinumtoxinA for the treatment of glabellar lines showed that no patients (0/105) developed neutralizing antibodies over the 84-day study period as tested by a fluorescence immunoassay followed by the MDA (Imhof and Kühne 2011).

### RimabotulinumtoxinB

RimabotulinumtoxinB is not approved for the management of glabellar lines in the United States, and no information is available on the immunogenicity rate in cosmetic applications.

## Future directions

Overall, the immunogenicity rates reported in the literature for all modern type A botulinum neurotoxin products are low. However, there are some limitations to these data, as many studies evaluate immunogenicity rates over the short term. Examination of longer treatment periods over all indications would help to ascertain the rates at which a patient is likely to develop secondary non-response and/or neutralizing antibodies over time. Furthermore, differences in assay sensitivity may lead to differences in reported rates of immunogenicity, and subsequently, rates cannot be directly compared among products. The use of a standardized assay or combination of assays to assess the presence of neutralizing antibodies may be beneficial. Head-to-head clinical trials also would facilitate comparison of the immunogenicity rates of the different products; however, such trials would be impractical due to the extremely large numbers of patients that would likely be required to show differences.

Additional research may resolve some of the controversies surrounding antibody formation and clinical effectiveness. A limitation of many studies is that only secondary non-responders were tested for the presence of antibodies, and thus limited information is available on the incidence of, or mechanism underlying, primary non-response to botulinum neurotoxin treatment. Clinicians may prevent and manage primary and secondary non-response to treatment in several ways. Doses should be as low and infrequent as possible to avoid or delay the development of neutralizing antibodies. Factors beyond immunogenicity that may contribute to non-response, such as technical issues and changes in disease state over time, should be considered. Finally, in the event of persistent non-response to treatment, where immunogenicity is considered the most probable explanation, a different botulinum neurotoxin serotype could be tried.

## Conclusions

Botulinum neurotoxin is usually employed as a treatment for chronic disorders and patients treated successfully with botulinum neurotoxin products may require continuing treatment over many years. Overall, the immunogenicity rate for all type A botulinum neurotoxins is low and the type B serotype formulation appears to be more immunogenic than the commercialized botulinum neurotoxin type A products. However, it should be noted that differences in factors such as assay sensitivity and specificity, sample handling, and underlying disease may lead to differences in reported rates of immunogenicity and may compromise comparisons of botulinum neurotoxin products. Treatment failure and secondary non-response to botulinum neurotoxin products are often the result of factors other than the presence of neutralizing antibodies. Nevertheless, in view of the potential risk for secondary treatment failure, clinical strategies to reduce or eliminate potential risk factors that may lead to the development of neutralizing antibodies are to be considered. At the present time an accepted strategy is to mitigate antibody formation using the lowest effective doses that produce a meaningful therapeutic effect and employing the longest inter-injection interval that is clinically acceptable.
